# Pupil dilation reflects the time course of emotion recognition in human vocalizations

**DOI:** 10.1038/s41598-018-23265-x

**Published:** 2018-03-20

**Authors:** Manuel Oliva, Andrey Anikin

**Affiliations:** Lund University, Cognitive Science, Lund, SE-22100 Sweden

## Abstract

The processing of emotional signals usually causes an increase in pupil size, and this effect has been largely attributed to autonomic arousal prompted by the stimuli. Additionally, changes in pupil size were associated with decision making during non-emotional perceptual tasks. Therefore, in this study we investigated the relationship between pupil size fluctuations and the process of emotion recognition. Participants heard human nonverbal vocalizations (e.g., laughing, crying) and indicated the emotional state of the speakers as soon as they had identified it. The results showed that during emotion recognition, the time course of pupil response was driven by the decision-making process. In particular, peak pupil dilation betrayed the time of emotional selection. In addition, pupil response revealed properties of the decisions, such as the perceived emotional valence and the confidence in the assessment. Because pupil dilation (under isoluminance conditions) is almost exclusively promoted by norepinephrine (NE) release from the locus coeruleus (LC), the results suggest an important role of the LC-NE system during emotion processing.

## Introduction

Imagine that you hear someone yelling in pain, or laughing: these emotional vocalizations may often carry no linguistic content, yet they convey immediate information about the emotional state of the speaker. When individuals are exposed to affective signals such as nonverbal emotional vocalizations, their pupils usually increase in size as the stimuli are perceived. These pupillary responses were therefore described as reflecting autonomic arousal triggered by emotional stimuli^1,2^. On the other hand, changes in pupil size are known to be linked to cognitive processing^3,4^ during tasks that do not involve emotional stimuli. Because recent evidence suggests that emotion processing recruits cortical regions normally associated with cognition^5–7^, in this study we investigate whether pupil responses can be used to betray the underlying process of affective processing.

Previous studies report that emotionally arousing stimuli, both auditory and visual, trigger bigger increases in pupil size than emotionally neutral stimuli^1,2^. Within emotional stimuli, some authors^8,9^ found that negatively valenced stimuli (e.g., crying) trigger larger pupil dilations than positive stimuli (e.g., laughter), whereas others found that both positive and negative stimuli could generate equally large pupil responses^1,2^. Despite some discrepancies, this evidence led to interpret pupillary responses as autonomic reactions elicited by arousing stimuli^1,2^ rather than as a reflection of cognitive emotional processing. This view is supported by findings showing that pupil dilation correlates with measures of arousal such as skin conductance^2^, and that stimuli portraying sexual content trigger especially large pupil responses^10,11^. However, emotional vocalizations are perceptual stimuli that require sensory integration in order to be decoded^7,12–14^, which may make it cognitively demanding to identify the emotional state of the speaker.

Apart from emotional stimuli, cognitively effortful tasks have long been known to influence pupil size^15^. Accumulating evidence shows that, under isoluminance conditions, changes in pupil size can be attributed almost exclusively to noradrenaline (NE) release from the locus coeruleus (LC)^16^. The LC is the main noradrenergic nucleus in the brain, which sends projections to several cortical regions. The LC is thus believed to exert wide brain modulation of behavioral decisions^3,4^. In addition, recent studies point out a correlated activity between noradrenergic and cholinergic activity in regulating neural states such as alertness^17^. This cholinergic activity was also shown to influence pupil dilation^18^. The link between neuromodulatory activity and pupil size allowed other studies to show associations between pupil dilation and performance in attentional tasks^19^, visual discrimination^20^, and the speed of visual perceptual choices^21^. For instance, a study reported that pupil dilation predicted the stability of decisions under perceptual rivalry^22^.

Perceptual decisions require the accumulation and integration of noisy sensory information, and therefore perceptual decisions usually develop gradually^23,24^. In a similar vein, people exposed to emotional vocalizations may entertain several possible interpretations of the emotional state of the speaker^25^. There is evidence that different brain regions process auditory emotional stimuli in a hierarchical fashion^6,13,26^. The listeners may then need to accumulate perceptual information in order to reach a decision about the emotion portrayed by the stimulus^7,12,14^. The processing of affective stimuli was first thought to be mediated by subcortical regions such as the amygdala. However, recent studies show that cortical areas have a more important role in emotion processing than it was previously thought^6,27^. In fact, current views of affective processing propose a dynamic interaction between cognitive and emotional areas that challenge functional boundaries^28,29^. As the perception of emotional states seems to depend on interactions between cortico-emotional regions, we expect that pupil dilation should reflect both dimensions of emotion processing.

In the present study, we investigate whether pupil responses reflect cognitive and attentional mechanisms inherent in emotion processing (in addition to simple autonomic responses to arousing stimuli, as previously reported). For this purpose, participants were exposed to naturalistic human vocalizations while they were eye-tracked under isoluminance conditions. In order to analyze the emotion selection process, participants were asked to indicate the moment when they had identified the emotional state of the speakers producing the vocalizations. Later, participants reported the perceived emotional valence of the vocalization and the confidence in their own assessment. We analyzed these data in order to ascertain whether the emotional selection process could be traced through changes in the pupil size of the listeners. Results suggest that pupil responses reflect the time course of affective processing, and that the intensity of perceived emotion only enhances pupil dilation, without being its main driver. We show that pupil dilations are not simply the product of emotional responses, but a rich source of information about affective processing, which opens new avenues for emotion processing research.

## Results

### Valence and confidence ratings

**Figure 1:**
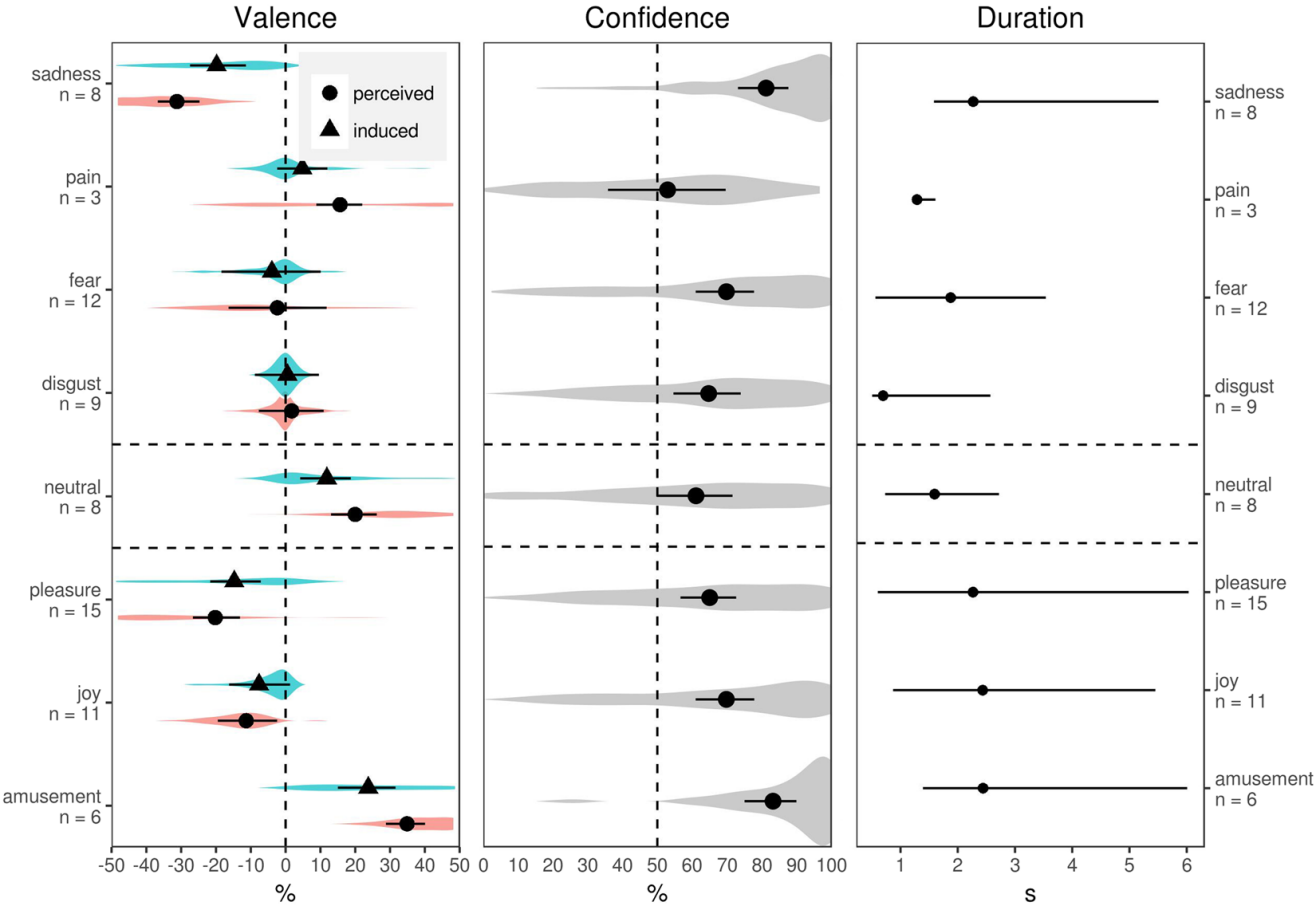
Valence of perceived and induced emotion, confidence ratings, and sound duration averaged per emotion category. For valence and confidence ratings, solid points show median fitted values with 95% CI, and violin plots show the distribution of answers. Median with range is shown for sound duration. The number of sounds in each category is listed underneath emotion labels.

In order to separate the contribution of affective and cognitive processes to pupil response, we aimed to test stimuli that varied in terms of the valence (positive/negative/neutral), intensity, and ease of recognition of the expressed emotion. As shown in Fig. 1, sounds of amusement and sadness offered unambiguous examples of highly positive and negative stimuli, respectively. For neutral stimuli, the valence of both perceived and induced emotion was close to zero (induced valence was estimated based on how participants rated their own emotional experience upon hearing the sound, rather than the experience of the speaker, see Methods for details). The remaining categories covered a wide range of positive, negative, and nearly neutral ratings, often with low confidence. Duration also varied both within and across emotional categories (Fig. 1). We therefore succeeded in developing an array of test sounds that varied in duration, valence, intensity, and ease of identification. This allowed us to assess the independent effects of all these factors on pupil response.

### Task-evoked pupil responses

**Figure 2:**
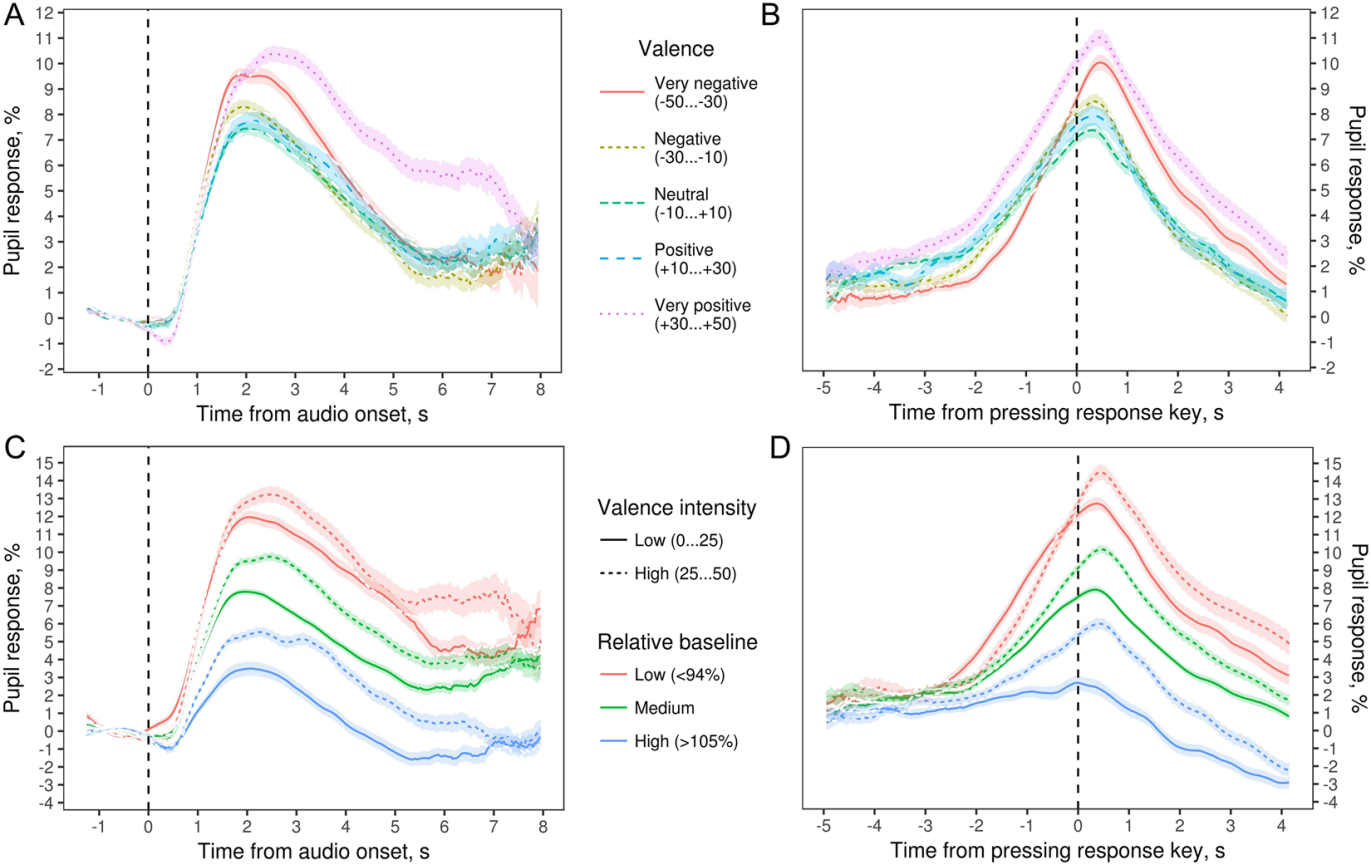
Aggregated pupil response as percentage change in pupil size compared to the pre-trial baseline. (**A**,**B**) Aggregated by valence and time-locked to either audio onset (**A**) or pressing the response key (**B**). (**C**,**D**) aggregated by valence intensity and the ratio of pre-trial baseline to resting baseline. As expected, emotionally charged sounds triggered larger pupil dilations in comparison to neutral sounds. Shaded areas represent standard errors for each time point.

We hypothesized that peak pupil response, defined as maximum post-presentation pupil dilation, would be greater for sounds with both very positive and very negative valence compared to relatively neutral sounds. Simple aggregation of pupil responses across all trials shown in Fig. 2 (panel A) appeared to bear out this prediction. Apart from valence, we expected pupil response to be affected by the confidence with which participants recognized the speaker’s emotion. In addition, sound duration may be affecting the shape of pupil response curves in Fig. 2 (panel A), since positive vocalizations were on average about 1 s longer than negative and 1.3 s longer than neutral sounds (Table 2). To tease apart the independent effects of these factors on peak pupil response, we fit multiple regression models, which evaluate several predictors simultaneously and estimate their independent effects. We modeled four different characteristics of pupil response curves: peak pupil dilation, the time of peak dilation, the rate of pre-peak dilation, and the rate of post-peak contraction. Together, they provide a complete summary of the overall shape of pupil response curves.

In addition, the amount of pupil dilation is also known to depend on baseline pupil size. In this regard, the relation between pre-trial baseline and pupil size at rest (when pupil is measured at passive fixation) was another factor that had a major effect on pupil response. The average ratio of pre-trial baseline to resting baseline was 0.99 ± 0.09 (M ± SD), and pupil response was apparently enhanced when the pre-trial baseline was low relative to the resting baseline and attenuated when their ratio was high (Fig. 2, panel C). This speaks in favor of controlling for baseline pupil size at rest, when participants were not engaged in any cognitive-emotional task (resting baseline). Therefore, we used mixed models to analyze raw pupil size with pre-trial baseline as a predictor (to account for pupil size at the beginning of each trial) and a random intercept for each participant (to account for individual variability in overall pupil size).

#### Peak pupil dilation

Controlling for pre-trial baseline, confidence, duration, and response time, peak pupil response did not depend on whether the perceived valence was positive or negative (*L* = 0.5, *df* = 1, *p* = 0.47). Similarly, the arrow key that participants pressed to indicate readiness to rate the sound (left = negative, right = positive) did not predict the size of peak pupil dilation (*L* = 2.1, *df* = 1, *p* = 0.15). In contrast, the absolute distance of perceived valence ratings from the neutral point in the middle of the scale, which we hereafter refer to as “valence intensity”, was a significant predictor of peak pupil response even after accounting for baseline, confidence, sound duration, and response time (*L* = 10.4, *df* = 1, *p* = 0.001). There was no interaction between valence intensity and its positive or negative “sign” as predictors of pupil response (*L* = 0.01, *df* = 1, *p* = 0.91). The pupil therefore responded to emotionally charged sounds in general, regardless of whether they were perceived as expressing positive or negative emotional states. We obtained similar results using ratings of induced rather than perceived valence (not shown). The ratings of perceived and induced valence were highly correlated (*r* = 0.81), so in the rest of analyses we focus on perceived valence.

The effects of pre-trial baseline, valence intensity, confidence, sound duration, and response time on peak pupil dilation were statistically significant when included in the same multiple regression model (*L* > 9.0, *p* < 0.002 for each; see Table 1). The effect sizes were rather small, however. Controlling for pre-trial baseline, confidence, sound duration, and response time, the pupil was predicted to dilate by an extra 0.07 mm (95% CI [0.03, 0.11]) in response to sounds of maximal vs. neutral valence intensity. The effect of increasing confidence from 0% to 100%, again controlling for the other variables in the model, was to attenuate peak pupil response by 0.07 mm (95% CI [−0.12, −0.03]; see also Supplementary Figure S2). Over the observed range of sound duration (0.5 to 6 s) and response times (0.6 to 9 s) these two predictors affected pupil response more powerfully than did ratings of confidence and valence intensity. In addition, the effect of pre-trial baseline on peak pupil response was an order of magnitude stronger than the effects of other predictors (see Table 1). However, it is worth reiterating that all these predictors were evaluated simultaneously using multiple regression, and the effect of valence intensity, confidence, sound duration, and response time remained significant after controlling for baseline pupil size.

**Table 1:** Predictors of peak pupil dilation and its timing.

Predictor (Range)	Pupil Dilation (mm)	Peak Time (s)	Dilation Rate (mm/s)	Contraction Rate (mm/s)
Valence Intensity (0…50)	0.07 [0.03, 0.11]	0.10 [−0.01, 0.21]	0.02 [0, 0.04]	−0.02 [−0.05, 0]
Confidence (0…100)	−0.07 [−0.12, −0.03]	−0.15 [−0.28, −0.02]	0.00 [−0.03, 0.02]	0.04 [0.01, 0.06]
Sound Duration (0.5…6 s)	0.22 [0.16, 0.27]	0.35 [0.22, 0.47]	−0.01 [−0.04, 0.01]	−0.04 [−0.07, −0.02]
Response Time (0.6…9 s)	0.20 [0.12, 0.27]	1.06 [0.85, 1.27]	−0.10 [−0.14, −0.06]	−0.03 [−0.07, 0.01]
Baseline (3.0…7.0 mm)	2.39 [2.29, 2.5]	−0.97 [−1.29, −0.65]	−0.38 [−0.44, −0.33]	−0.33 [−0.43, −0.24]
Peak Dilation (3.6…7.6 mm)	—	—	—	−0.02 [−0.05, 0]

Beta-coefficients in linear mixed-effects regression: the median of posterior distribution and 95% credible intervals.

To summarize, the pupil responded more vigorously to auditory stimuli of higher emotional intensity, longer duration, and higher ambiguity (measured by slower responses and lower confidence in the nature of perceived emotion). This variation in pupil response was relatively subtle compared to the major impact of baseline pupil size, but it was nevertheless highly significant and allowed us to separate the individual contributions from cognitive (confidence, response time) and affective (valence intensity) components. This suggests that both affective processing and cognitive load make independent contributions to pupil dilation.

#### Time of peak dilation

In addition to the magnitude of peak pupil dilation, we also investigated its timing. The effects of valence intensity, confidence, sound duration, and response time on peak time were consistent with their effects on peak height (compare the first and second columns in Table 1). The peak of pupil response was delayed when participants heard emotionally charged rather than neutral sounds, albeit only marginally (+0.1 s, 95% CI [−0.01, 0.21] for maximum versus neutral valence intensity). Sound duration and response time also predicted later peak dilation. In contrast, higher confidence predicted a peak that was 0.15 s earlier (95% CI [0.02, 0.28]) for very high versus very low confidence. As expected, higher pre-trial pupil size was associated with an earlier peak, as pupil dilation may face ceiling effects related to maximal dilation.

#### Rate of pre-peak dilation

Another important characteristic of pupil response curves is their slope. To test which factors affected the rate of pupil dilation, we fit a multiple regression model for predicting the ratio of peak height to peak time. Since ratios are strongly affected by outliers, especially small values in the denominator, we excluded 23 trials with peak time under 0.25 s (~1% of data). Controlling for other predictors in the model, slower response times also slowed down pupil dilation by −0.10 mm/s over the entire range of observed response times, 95% CI [−0.14, −0.06]. Likewise, pre-trial baseline also had a significant negative effect on the pupil dilation rate (−0.38 mm/s, 95% CI [−0.44, −0.33]), indicating that lower baseline levels were followed by faster pupil dilation. Finally, the pupil dilated slightly faster when the sound had high vs. neutral valence intensity: 0.02 mm/s [0, 0.04]. Confidence and sound duration did not affect the rate of pupil dilation (see Table 1, column 3).

#### Rate of post-peak contraction

We analyzed the rate of pupil contraction over 2 s from the moment when the pupil reached its maximum size. The time of 2 s corresponded to the moment when the pupil on average contracted to about 50% of its peak diameter, and it also allowed us to preserve 95% of trials (eye tracking was terminated 4.5 s after the participant pressed the response key, and therefore the duration of available post-peak pupil size data varied across trials). We included an additional predictor in the model for post-peak contraction, namely the magnitude of peak dilation, as the relation between the speed of dilation and contraction may as well be related to the magnitude of peak pupil dilation. Controlling for other predictors, the pupil contracted faster in trials with higher confidence:+0.04 mm/s, 95% CI [0.01, 0.06]. The rate of contraction was attenuated by −0.04 mm/s (95% CI [−0.07, −0.02]) for longer sounds, presumably because continuing exposure to the stimulus reduced the speed with which the pupil size returned to baseline. Valence intensity, baseline pupil size, and peak height all slowed down post-peak pupil contraction, whereas response time had no effect on the rate of contraction (see Table 1, column “Post-peak contraction”).

### Temporal alignment of behavioral and pupil response

**Figure 3:**
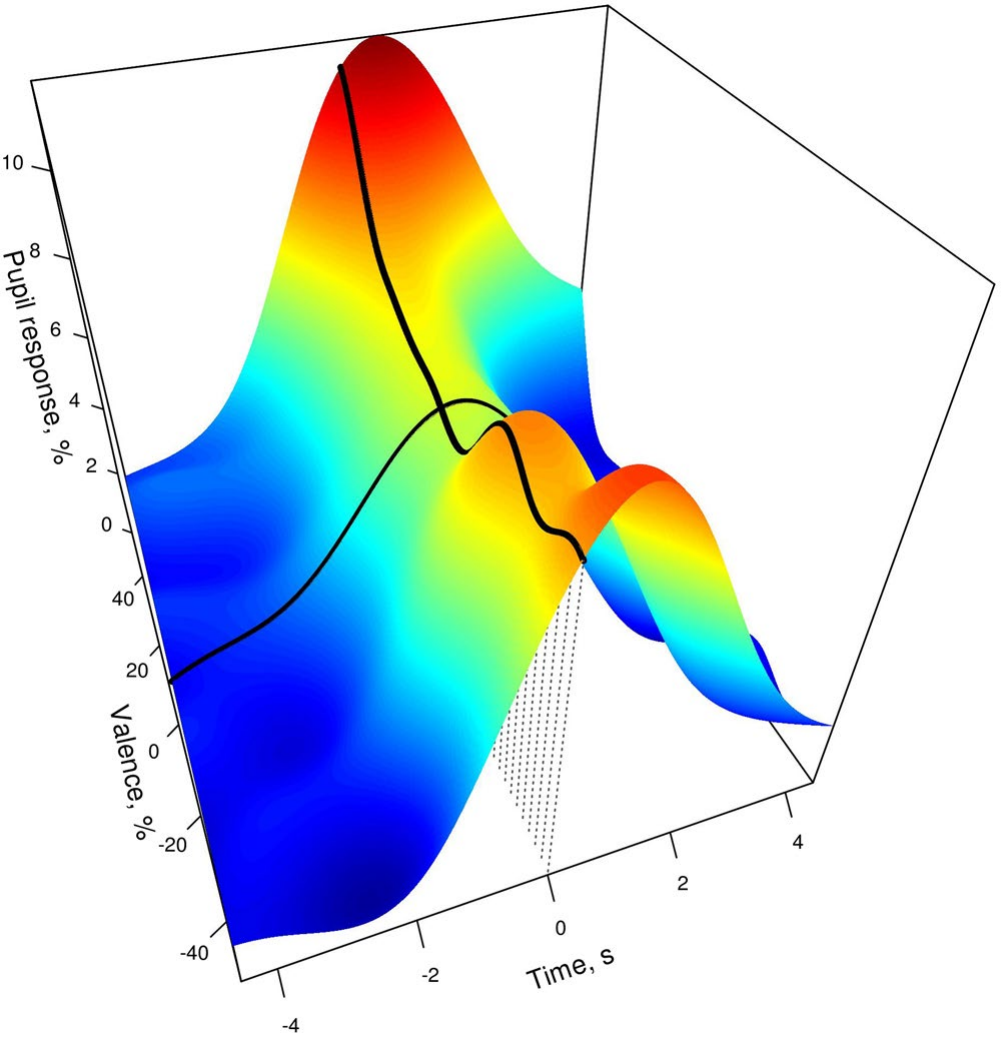
Pupil response relative to baseline time-locked to pressing the response key. Generalized additive model with a single smooth term for a combination of time and emotion rating and a linear term for trial baseline. Elevation and color show the size of pupil dilation compared to the baseline. The “valence intensity” coordinate shows the rating of the speaker’s emotion given after pressing the response key (time 0): −50 = very negative, 0 = neutral, +50 = very positive. The thin black line traces pupil trajectory for a neutral sound. The thick black line and the dotted lines mark the moment of response.

When analyzing pupil response to emotionally charged stimuli, an interesting question is how the time of maximum pupil dilation relates to the time of decision making. In this study, participants pressed one of the two response keys - left for negative, right for positive - indicating readiness to rate the caller’s emotion. To separate the components of pupil response before and after making this decision, we extracted pupil response curves time-locked to pressing the response key. As shown in Fig. 3, the moment of response slightly preceded peak pupil dilation: the average time from pressing the response key to reaching maximum pupil dilation was 0.37 ± 1.7 s (M ± SD).

Participants tended to respond 1–2 s after sound offset if the sound was very short, whereas for sounds longer than about 3 s they tended to respond while the sound was still playing, with a lot of individual variation (see Supplementary material Figure S1). Interestingly, even though participants responded more slowly to longer sounds (effect of duration on arrow response time: *L* = 21.2, *df* = 1, *p* < 0.001), sound duration had no effect on the lag between peak pupil dilation and pressing the response button (*L* = 0.06, *df* = 1, *p* = 0.81). Figure 3 suggests that this lag is more pronounced for both very negative and very positive sounds compared to neutral sounds. This is confirmed by regression analysis: both the valence intensity of perceived emotion and confidence are independent positive predictors of the lag between peak pupil response and pressing the response key (*L* = 6.0 and 5.3, *p* = 0.01 and 0.02, respectively, *df* = 1 for both). However, this effect is both uncertain and relatively weak: the time from response to peak pupil dilation is predicted to be 280 ms greater (95% CI[10, 550]) for sounds of maximal vs. neutral valence intensity and 360 ms greater (95% CI[8, 560]) for high vs. low confidence. This indicates that lower cognitive effort and higher perceived emotional intensity push peak pupil response slightly beyond the moment of decision making.

To summarize, peak pupil response was delayed and enhanced for longer versus shorter sounds, but it still closely coincided with the moment when a participant made up their mind about the nature of portrayed emotion and pressed the response key (see Fig. 3). This speaks in favor of analyzing pupil response curves time-locked to response, rather than to onset of stimulus presentation, since this allows us to abstract from stimulus duration.

## Discussion

In the present study we measured pupil responses while participants formed decisions about the emotional state of speakers. For this purpose we used nonverbal vocalizations that portrayed several emotional states of varying intensity and ambiguity^25^. This investigation is the first to show that pupil responses to emotionally charged stimuli are temporally aligned with decision making about the nature of the speaker’s emotion. Additionally, the results indicate that pupil dilation predicts properties associated with the listeners’ choices, such as the perceived emotional valence of the stimulus and the confidence level in their own judgment. Specifically, peak pupil responses were larger for stimuli rated with high valence intensity and when the listener was less confident about the stimulus valence. Previous experiments^30,31^ have shown that when an individual’s cognitive system is taxed during emotion processing (e.g., with a dual-task manipulation), pupil dilation and other physiological measures can reflect contributions from both cognitive effort and emotional arousal. In contrast, here we show that the task of emotion recognition in itself is sufficient to produce a marked pupil response, the time course of which reflects the attentional and cognitive involvement inherent in the decoding of the respective affective signals.

This is also the first study that assessed pupil responses to naturalistic emotional vocalizations, recorded in real-life situations, associated with a wide range of affective states, and varying in valence, intensity, ambiguity, and duration^25^. In line with previous reports^1^, we found that the perceived emotional valence of the sound caused larger pupil dilations in comparison to sounds perceived as neutral. However, the emotional valence seemed to have only a minor contribution to the overall pupillary response. Emotionally salient sounds did trigger larger dilations, but this effect was relatively small (2%) in comparison to both the overall pupil responses (10%) and the effect of sound duration and response time (∼3–5%). In addition, sounds perceived as neutral also triggered pupil responses that significantly separated from baseline (see Fig. 2), indicating that pupil dilation did not exclusively depend on the perceived emotional valence. In contrast, pupil fluctuations seemed to follow the decision formation about the emotional state of the speaker. In particular, the pupil size started to respond shortly after audio onset and continued to dilate throughout the emotional state selection process. This sustained increase in pupil size continued until just after the decision about the emotional valence was made, following which pupil size returned to baseline levels (see Figs. 2 and 3). Such pupillary behavior was consistently robust across a wide range of stimuli that varied in emotional valence, ambiguity, and duration. Taken altogether, the results indicate that the time course of pupil response betrays the perceptual process of selecting the emotional state of the speaker, whereas the perceived emotional valence intensity remains only as a moderator of the response.

Current views of visual perceptual decision making propose that when there is a need to select one of several competing perceptual options, individuals must accumulate and integrate perceptual evidence in order to select between alternatives^23,32^. In a similar fashion, the decoding of nonverbal vocalizations is not always a perceptually trivial task, and vocalizations can often be interpreted in different ways. Naturalistic vocalizations can be very intense, but inherently more ambiguous than actor portrayals of particular emotions, which are intended to be maximally transparent^25^. This reduced correlation between emotional intensity and ease of recognition allowed us to disentangle their independent effects on pupil response. We propose that the sustained increase in pupil dilation at the beginning of trials reflects this period in which perceptual evidence is decoded and integrated in order to decide between competing perceptual alternatives, which demands cognitive resources. As sufficient information is gathered, individuals can select the most likely emotional state of the speaker. After a selection is made, there is no need to sustain high cognitive engagement in processing the vocalizations, which in turn leads to a decrease in pupil size. In this process, vocalizations perceived as ambiguous demand more cognitive resources and take more time before they are fully decoded, which translates into larger pupil dilations. In support of this interpretation, we observed that peak pupil dilation was delayed and enhanced when the confidence ratings were low. In addition, low confidence caused the pupil to contract more slowly (after peak dilation), probably reflecting prolonged processing of the stimuli.

There is still disagreement about whether positive and negative stimuli trigger equally large pupil responses. Whereas some studies reported that negatively valenced emotions trigger larger pupil dilations than positively valenced ones^8,9^, some others found that both positive and negative stimuli can trigger equally large dilations^1^. In line with the latter view, we found that the emotional modulation of the pupil response was independent of whether the stimuli were perceived as positive or negative. The peak amplitude of pupil responses was better predicted by the stimulus valence intensity than its valence (positive or negative). In other words, both positive and negative emotional stimuli were capable of causing equally large pupil responses; however, the more a stimulus deviated from an emotionally neutral sound, the more pupil dilation it elicited.

Although the mechanisms of auditory emotional processing are not fully understood, recent studies propose that these emotional signals are processed in a multi-step, hierarchical fashion, involving both subcortical (e.g., amygdala) and cortical regions (e.g., prefrontal cortex)^6,7^. Brain structures were normally functionally separated into areas that process emotion (e.g., the amygdala) and those related to cognition (e.g., prefrontal cortex). However, recent evidence challenges the idea of division between emotion and cognition as excluded from each other. Different studies demonstrate that, for instance, cortical areas show activation patterns to emotional stimuli that are as, or even more, consistent as that of the amygdala^6,26^. Conversely, arousal responses are equally critical for cognitive functions^33,34^. For instance, arousal responses that involve the amygdala and the LC are important for memory consolidation^35^ and attention^33,34,36,37^. Because we find that pupil size is sensitive to both cognitive and emotional factors, pupil dilation may open new research avenues for the study of cognitive-emotional interactions.

Under isoluminance conditions, pupil dilation primarily reflects noradrenergic^16^ and cholinergic neuromodulation^17^. In particular, some studies suggested that pupil baseline tracks the tonic activity state of the LC-NE^3^. Here, we found that pupil baseline was a significant predictor of peak dilation as well as of pupil dilation rate in response to affective processing. In a similar vein, different studies found associations between pupillary responses and mental effort^15^, visual perception^20,38^ and the speed of visual discrimination tasks^21^. These previous studies, however, used mostly visual tasks that did not include emotional processing. Thus, here we extend such results to perceptual discrimination of affective stimuli, where pupil dilation also reflects the processing of emotional signals. In a schema of cognitive-emotion interplay, our results suggest that the LC, with its high connectivity (e.g., amygdala, prefrontal cortex), emerges as one potential hub that integrates emotional and cognitive inputs during affective information processing.

## Methods

### Participants

Thirty-three university students (mean age = 25 years, SD = 2.8, age range =21–35 years; 13 men) voluntarily participated in the experiment and received a cinema ticket in return. No participant suffered from significant data loss (see Data Analysis), and all participants were included in the analysis.

### Ethics statement

In accordance with the Swedish law (SFS 2003: 460, 16 §), all participants gave written consent for taking the experiment. In accordance with the Swedish Act concerning the ethical review of research involving humans (2003:460), the present study was exempt from the requirement for ethical approval.

### Setup

The study was conducted in a room equipped with remote eyetracking systems. The auditory stimuli were presented through headphones connected to a computer and a 22-inch monitor (DELL P2210, 1680 × 1050 at 60 Hz). A viewing distance of approximately 65 cm was maintained using chinrests. The pupil diameter of the participant’s right eye was measured by a noninvasive infrared “RED-m’’ (SMI, Teltow, Germany) eye tracker at a rate of 120 Hz. All visual and auditory stimuli were presented using Psychopy^39^ (Version 2.85) and SMI iView X (2.8.43).

### Stimuli

Experimental stimuli consisted of 72 sounds, including 68 authentic emotional non-linguistic vocalizations from a validated corpus^25^ and four neutral stimuli from Montreal Affective Voices^40^ (MAV). The neutral sounds from MAV contained a single vowel [a] pronounced with a flat intonation. The authentic emotional vocalizations consisted of a variety of laughs, screams, moans, grunts, and other sounds obtained from real-life video footage on social media. The accuracy with which particular emotions were recognized by listeners was reported in the original study^25^, allowing us to select both unambiguous (n = 36) and ambiguous (n = 32) sounds. We were less interested in the accuracy of discriminating individual emotional states such as pain and disgust, so our selection criterion of ambiguity involved only the accuracy of recognizing the valence as positive or negative in the validation study^25^. We also strove to include sounds of varying intensity in both the ambiguous and the unambiguous groups. In particular, some ambiguous stimuli could be interpreted as either highly positive or highly negative. All sounds were normalized for peak amplitude to standardize their loudness^25^. Although loudness normalizations by either peak or root mean square amplitude are standard procedures in acoustic research, the cochlear frequency response curve is not flat^41^ and, as such, the spectral composition of a sound may influence its subjective loudness. Since loudness is known to affect the peak amplitude of pupil dilation^42^, this potential confound may need to be controlled more effectively in future studies.

The main characteristics of experimental stimuli are summarized in Table 2. Emotion categories in the left column correspond to the emotion most commonly perceived by listeners in the validation study^25^, rather than to the production context or the “true” underlying emotion of the speaker. Our objective in selecting these stimuli was to include a wide range of sounds that varied in their perceived valence (positive, negative, and neutral), intensity (from mild to extreme, as for uncontrolled sobbing or orgasmic moans), and ease of recognition. We verified that the selected positive and negative vocalizations were not significantly different in terms of intensity (*F*(1, 62) = 0.05, *p* = 0.82) and ease of valence recognition (*F*(1,62) = 1.88, *p* = 0.18). However, positive vocalizations were approximately 1 s longer compared to negative vocalizations (*F*(1, 62) = 8.2, *p* = 0.006). To account for this difference, we included duration as a covariate in all analyses.

**Table 2:** Description of experimental stimuli (N = 72).

Emotions	N stimuli (M/F)	Valence Recognition (%, M ± SD)	Perceived Intensity (%, M ± SD)	Duration (S)	Examples
Amusement	6 (2/4)	94 ± 15	58 ± 16	3.4 ± 1.9	Laughs
Joy	11 (4/7)	80 ± 20	40 ± 11	2.5 ± 1.4	Laughs, whoops
Pleasure	15 (8/7)	66 ± 19	49 ± 23	2.8 ± 1.9	Moans, whimpers
**Total Positive**	**32**	**76** ± **21**	**48** ± **19**	**2.8** ± **1.7**	—
**Total Neutral**	8*	—	—	**1.5** ± **0.7**	—
Disgust	9 (6/3)	86 ± 15	40 ± 20	1.1 ± 0.7	Grunts, sighs
Fear	12 (6/6)	77 ± 26	50 ± 22	1.7 ± 0.9	Screams, roars
Pain	3 (1/2)	50 ± 17	22 ± 6	1.4 ± 0.2	Gasps, whimpers
Sadness	8 (2/6)	94 ± 12	67 ± 15	2.9 ± 1.3	Cries
**Total Negative**	**32**	**81** ± **22**	**49** ± **23**	**1.8** ± **1.1**	—

The ratings are based on a previous validation study^25^. *Four neutral sounds from Belin *et al*.^42^ and four mild, hard-to-recognize sounds from Anikin & Persson^25^.

### Procedure

Participants received a brief oral description of the experiment, and subsequent instructions were all presented on the computer’s screen. Participants completed eight practice trials, in which they heard three negative, two neutral and three positive sounds. This allowed participants to familiarize themselves with the range of stimuli that they would later rate. After the practice trials, the eyetrackers were calibrated. After calibration a pre-experiment pupil resting baseline was recorded for 45 s. Participants commenced each trial by looking inside a fixation circle subtending 1.1° at the center of the screen and pressing the upper arrow key to indicate readiness to begin. In order to avoid temporal predictability, the sound stimulus began after a non-ageing foreperiod^43^ of 5–11 seconds, where the probability of it terminating per unit of time was constant after 5 seconds and curtailed at 11 s. The relatively long foreperiod provided enough time following each trial for the pupil to subside back to baseline. As the sound stimulus was played, participants first rated the perceived valence of the sound as either positive (right arrow key) or negative (left arrow key); they were instructed to respond as soon as they recognized the emotion of the sound. In order to record the peak pupil response, the eyetracking recording continued for 4.5 s after the participant’s manual response.

Participants were instructed to look inside the fixation circle until the end of the trial to ensure no gaze displacement. After this period the fixation circle disappeared, and participants received three questions about the sound stimulus. First, they had to rate the perceived emotional valence of the sound using a continuous rating scale, “Is the person experiencing positive or negative emotions?”. This question corresponds with the manual response they had to perform first by pressing one of two arrow keys, except that this time the rating was continuous rather than binary. Secondly, they stated their confidence about their assessment. The valence of induced emotional state was measured by the third question, “How do you feel about the sound? Does it convey positive or negative emotions?’’. The inclusion of this subjective report was intended to investigate whether the pupil response related more to the capacity to recognize the emotional state of the speaker or to the subjective experience of the listener. Participants rated each sound on a scale of 0 to 100, where 50 corresponded to a neutral sound. To make the results more intuitive, we present valence ratings on a transformed scaled: −50 = very negative, 0 = neutral, and 50 = very positive.

The experiment used 72 sound stimuli presented in two blocks, each of them containing an equal number of positive and negative sounds. The presentation order of the stimuli was randomized for every participant, and they could take a break before starting with the second block.

### Data Analysis

Resting baseline was obtained by means of recording the pupil diameter as each participant passively looked at a fixation point for one minute before hearing any experimental stimuli. In addition, a pre-trial baseline was obtained before each trial. It was calculated as the average pupil diameter over a period of 1.3 s before sound onset (at the end of the intertrial foreperiod). Pupil size was recorded until 4.5 s after the participant’s response (left/right arrow key press). Pupil diameter data were pre-processed in Python (2.7.11) to detect and remove blinks and gaze displacement. Trials in which their gaze was displaced from the fixation circle were excluded from the analysis, because it can affect pupil diameter measurement. Periods of blinks were completed using cubic spline interpolation. For computational efficiency, pupil signals were then smoothed using a Savitsky-Golay filter (window = 21, order = 3) and downsampled from 120 Hz to 30 Hz. Trials where blinks and missing data exceeded 20% of the total trial samples were considered invalid and excluded from all analyses. We excluded 5.2% of all trials, preserving on average 68 out of 72 trials per participant (median 70, range 41 to 72). No participants were excluded from the analysis.

All statistical analyses and plotting were performed in R 3.4 (https://www.r-project.org). To analyze the valence and confidence ratings for each emotion, which we recorded on a scale from 0 to 100%, we assumed that the outcome variable followed a beta distribution and applied Bayesian beta regression. The valence of both perceived and induced emotion was analyzed within a single mixed model with an interaction term Emotion x Type of valence (perceived or induced) and two random intercepts: per participant and per stimulus.

The location and height of peak pupil dilation, as well as the rate of change in pupil size before and after the peak, were extracted from the pupil response curve for each trial. These four measures were then analyzed using Gaussian linear mixed models with pre-trial baseline as a predictor (to account for pupil size at the beginning of each trial) and a random intercept for each participant (to account for anatomical differences in pupil size and overall level of arousal). This allowed us to work with unaggregated trial-level data, while taking into account the variability of pupil response due to differences between participants and between experimental sounds. Statistical significance of predictors was tested with likelihood ratio tests using lme4 package^44^. To extract confidence intervals, we fit analogous Bayesian models, which arguably offer more robust estimates in the context of multilevel regression. All Bayesian models were created in Stan computational framework (http://mc-stan.org/) accessed with brms package^45^. To improve convergence and guard against overfitting^46^, we specified mildly informative conservative priors.

Pupil response curves were also aggregated per category and analyzed as time series, as follows. Individual pupil response curves from each trial belonging to a particular category - for example, with particular valence intensity ratings or baseline pupil size - were temporally aligned (time-locked to sound onset or pressing the response key) and added up. For each time point, we then calculated the standard error of the mean for pupil sizes measured at this time point across all participants and trials included in this category. In addition, we treated valence as a continuous variable and fit a Generalized Additive Model (GAM) from mgcv package^47^, specifying a single smoothing term for time and valence. This allowed us to visualize pupil response over time as a function of the perceived valence of the stimulus.

Python and R scripts for data preprocessing and statistical analysis, experimental sounds, and raw data are available in supplementary materials.

## Electronic supplementary material

Online Supplementary Material

Supplementary Information
