# Supplementary material for: Pupil dilation reflects the time course of emotion recognition in human vocalizations
Source: Sci Rep. 2018 Mar 20;8:4871. doi: 10.1038/s41598-018-23265-x (PMC5861097; doi:10.1038/s41598-018-23265-x)
Supplement: Supplementary file 1 — Online Supplementary Material [file 41598_2018_23265_MOESM1_ESM.pdf]

Online Supplementary Materials for:

Pupil dilation reflects the time course of emotion recognition in human vocalizations

M. Oliva & A. Anikin

Lund University, Cognitive Science

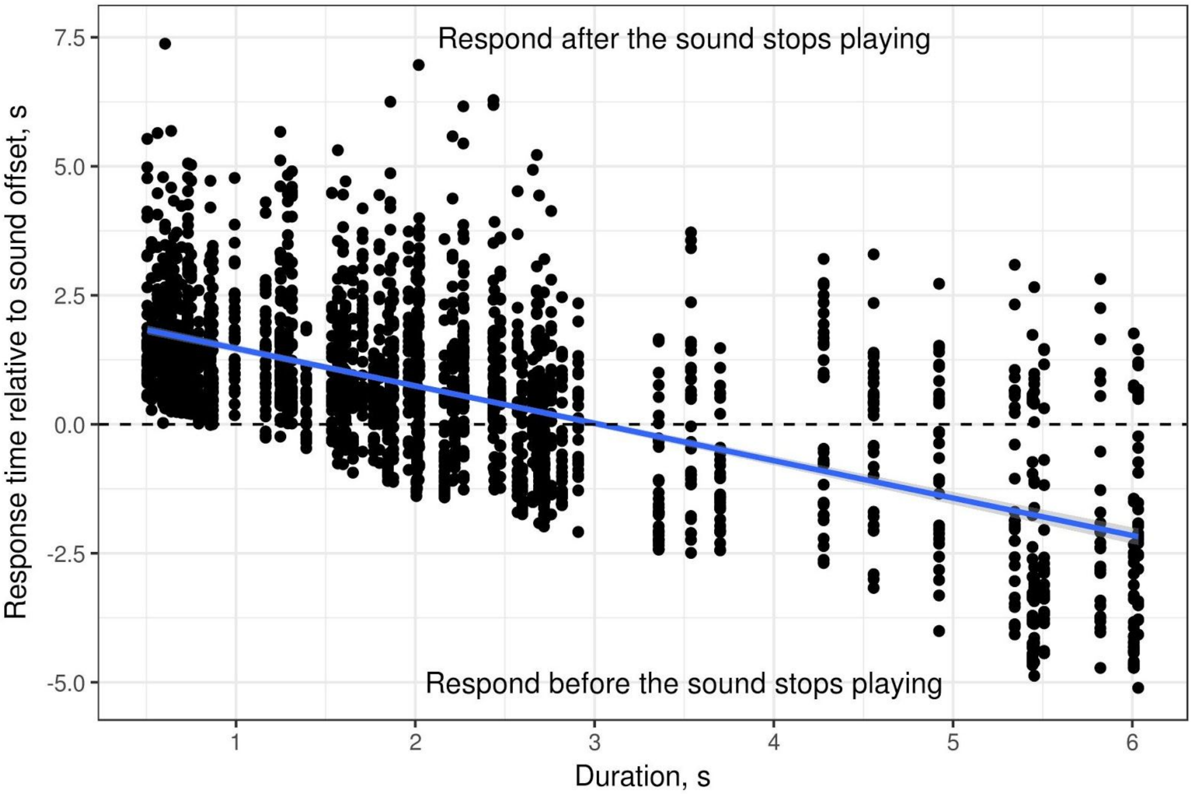

Figure S1. The difference between response time and sound offset as a function of sound duration. Participants tended to respond 1-2 s after sound offset if the sound was very short, whereas for sounds longer than about 3 s they tended to respond while the sound was still playing, with a lot of individual variation.

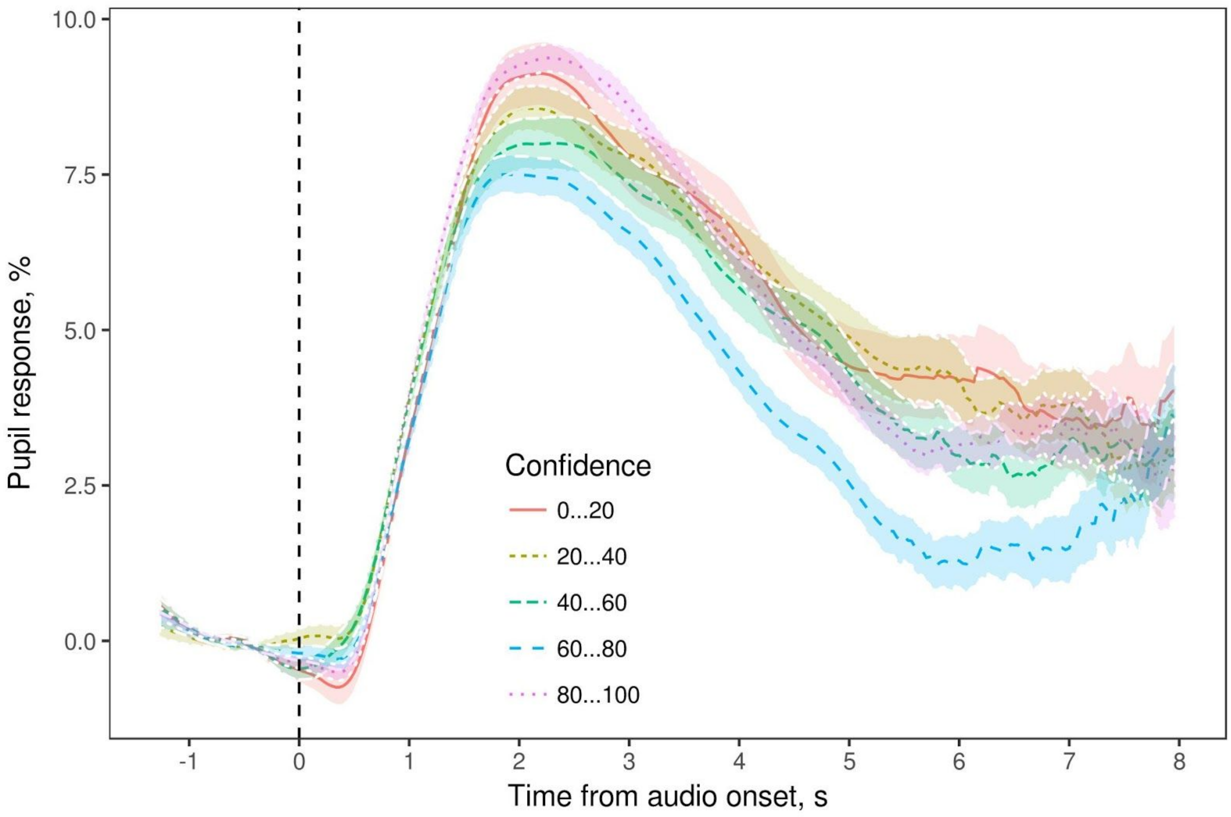

Figure S2. Pupil response curves aggregated from trials in which participants recognized the speaker's emotion with different degrees of confidence. Pupil response is enhanced for low-confidence sounds. There is an increase in pupil response for sounds with very high confidence (80% to 100%, ~41% of trials). The reason is presumably that high-intensity sounds also tended to be rated with very high certainty, and simple aggregation fails to distinguish between the contribution of these two factors.
